# A Targeted Mass Spectrometric Approach to Evaluate the Anti-Inflammatory Activity of the Major Metabolites of *Foeniculum vulgare* Mill. Waste in Human Bronchial Epithelium

**DOI:** 10.3390/molecules30071407

**Published:** 2025-03-21

**Authors:** Maria Assunta Crescenzi, Hector Gallart-Ayala, Cristiana Stellato, Ada Popolo, Julijana Ivanisevic, Sonia Piacente, Paola Montoro

**Affiliations:** 1 Department of Pharmacy, University of the Study of Salerno, Via Giovanni Paolo II 132, I-84084 Fisciano, Italy; mcrescenzi@unisa.it (M.A.C.); apopolo@unisa.it (A.P.); piacente@unisa.it (S.P.); 2 Ph.D. Program in Drug Discovery & Development, Department of Pharmacy, University of the Study of Salerno, Via Giovanni Paolo II 132, I-84084 Fisciano, Italy; 3 Metabolomics Unit, Faculty of Biology and Medicine, University of Lausanne, 1015 Lausanne, Switzerland; hector.gallartayala@unil.ch (H.G.-A.); julijana.ivanisevic@unil.ch (J.I.); 4 Department of Medicine, Surgery and Dentistry “Scuola Medica Salernitana”, University of Salerno, I-84084 Salerno, Italy; cstellato@unisa.it

**Keywords:** cell metabolomic, inflammation, vegetable waste

## Abstract

Fennel waste is rich in compounds that may have beneficial effects on human health. For this reason, the most abundant metabolites in fennel were isolated as the following: quercetin-3-*O*-glucoside, quinic acid, 1,5-dicaffeoylquinic acid, kaempferol-3-*O*-glucuronide, and quercetin-3-*O*-glucuronide. After inducing inflammation in human bronchial epithelial cells by stimulating them with IL-1β, the cells were treated with the specialized *Foeniculum vulgare* metabolites at different concentrations to assess their anti-inflammatory effect. Eicosanoids, fatty acids, and sphingolipids were extracted from the cell medium and quantified by UPLC-ESI-QTRAP-MS/MS analysis. The anti-inflammatory activity of the metabolites isolated from fennel waste was demonstrated. They were able to alleviate the inflammatory state in human bronchial epithelium by modulating the metabolic expression of both pro- and anti-inflammatory eicosanoids, fatty acids, and sphingolipids. These findings suggest the potential use of fennel waste in the production of dietary supplements to alleviate the symptoms of chronic inflammatory diseases like asthma, chronic obstructive pulmonary disease (COPD), and idiopathic pulmonary fibrosis (IPF), where the continuous use of antiphlogistics may have significant side effects.

## 1. Introduction

Cell metabolomics can provide deeper insights into the role of plant bioactive compounds in preventing and protecting against various diseases by modulating different metabolic pathways. The assessment of the biological effects of specific phytochemicals through in vitro metabolomic approaches can contribute to a more precise definition of metabolomic signatures and a better understanding of disease mechanisms in response to secondary metabolite exposure.

Cell metabolomics underscores the potential clinical impact of plant-based foods on prevalent diseases, suggesting that bioactive compounds derived from plants play a significant role, particularly in neurodegenerative diseases, cancer, and chronic conditions [1].

A diet rich in fruits and vegetables can modulate inflammatory states both in the elderly and in individuals affected by conditions characterized by chronic inflammation [2]. Furthermore, major health organizations recommend a vegetable-rich diet to prevent chronic diseases and promote overall health [3]. Specialized metabolites produced by plants benefit human health, including anti-inflammatory activity. Many natural compounds inhibit leukotriene biosynthesis by modulating the arachidonic acid pathway. For instance, Ramanan et al. demonstrated that 3-aryl-isocoumarin derivatives inhibit enzymes in pathways leading to the formation of leukotrienes and prostaglandins, thereby exerting anti-inflammatory effects [4,5].

The respiratory system is affected by numerous inflammatory diseases. Acute viral respiratory infections are a major cause of global mortality, either alone or in combination with secondary bacterial infections. While most viral respiratory infections are mild and resolve spontaneously, some can be more severe. Lower respiratory tract infections, such as bronchiolitis, croup, pneumonia, and exacerbations of asthma or chronic obstructive pulmonary disease (COPD), can lead to respiratory failure, severe health complications, death, and long-term cardiovascular or neurological consequences [6].

Eicosanoids are a class of metabolites derived from polyunsaturated fatty acids (PUFAs). They act as key mediators of inflammatory processes, and alterations in the levels of pro-inflammatory eicosanoids are associated with various pathologies, particularly those affecting the cardiovascular, gastrointestinal, and pulmonary systems [7,8]. Eicosanoids can be synthesized through the cyclooxygenase (COX) pathway, which leads to the production of prostaglandins, prostacyclins, and thromboxanes. Additionally, the lipoxygenase (LOX) pathway catalyzes the formation of certain eicosanoids, including leukotrienes and specific oxygenated derivatives at the C5 position, such as 5-hydroxy-6,8,11,14-eicosatetraenoic acid (5-HETE) and 5-oxo-6,8,11,14-eicosatetraenoic acid (5-oxo-ETE) [9]. In contrast, isoprostanes, another class of lipid mediators, are not enzymatically produced but instead arise from the free-radical peroxidation of arachidonic acid.

Sphingolipids are another class of inflammatory mediators. They are derived either from the metabolism of sphingomyelin, a major component of the plasma membrane, or through de novo synthesis. Several lipid mediators are enzymatically generated and play key roles in various cellular processes, including proliferation, apoptosis, and migration. Among the most important sphingolipid mediators involved in inflammation are ceramide, ceramide-1-phosphate (C1P), and sphingosine-1-phosphate (S1P). These molecules contribute to inflammatory responses by activating pro-inflammatory transcription factors in different cell types and inducing cyclooxygenase-2 (COX-2) expression, which subsequently promotes the production of pro-inflammatory prostaglandins [10,11].

Since secondary metabolites may exhibit anti-inflammatory activity, this study aimed to evaluate the ability of certain metabolites derived from fennel waste to modulate the eicosanoid and sphingolipid pathways. In particular, the plant metabolites selected for this cell metabolomics approach were those that had demonstrated the highest antioxidant and anti-inflammatory potential in preliminary spectrophotometric assays, as previously published [12]. Specifically, this research focused on assessing the anti-inflammatory potential of natural compounds derived from plant waste in inflammatory diseases of the respiratory tract. Building on previous chemical studies, we investigated the anti-inflammatory effects of these bioactive compounds in vitro. Human bronchial epithelial cells (BEAS-2B cell line) were treated with quercetin-3-*O*-glucoside, quercetin-3-*O*-glucuronide, kaempferol-3-*O*-glucuronide, 1,5-dicaffeoylquinic acid, and quinic acid, all isolated from fennel waste.

Inflammation was induced by stimulating the cells with IL-1β, after which cell culture media were collected for metabolite extraction and purification. Eicosanoids and sphingolipids were then quantified using UPLC-ESI-QTRAP-MS/MS analysis in multiple reaction monitoring (MRM) mode.

The anti-inflammatory activity of natural substances has been extensively studied, primarily through a transcriptomic approach [13,14]. This research proposes an innovative strategy for investigating the mechanisms of action of natural products in cellular systems by employing a metabolomic approach. To achieve this, a quantitative mass spectrometry method was developed to analyze molecules involved in inflammatory processes.

This study demonstrated that fennel waste, due to its high content of polyphenolic compounds, can reduce inflammation in the human bronchial epithelium by promoting the production of anti-inflammatory mediators while simultaneously inhibiting the production of pro-inflammatory mediators. These findings suggest that functional foods or nutraceutical products derived from fennel waste could serve as potential therapeutic strategies for managing chronic inflammatory respiratory diseases, such as chronic obstructive pulmonary disease (COPD) and asthma.

## 2. Results

### 2.1. Isolation

**Figure 1:**
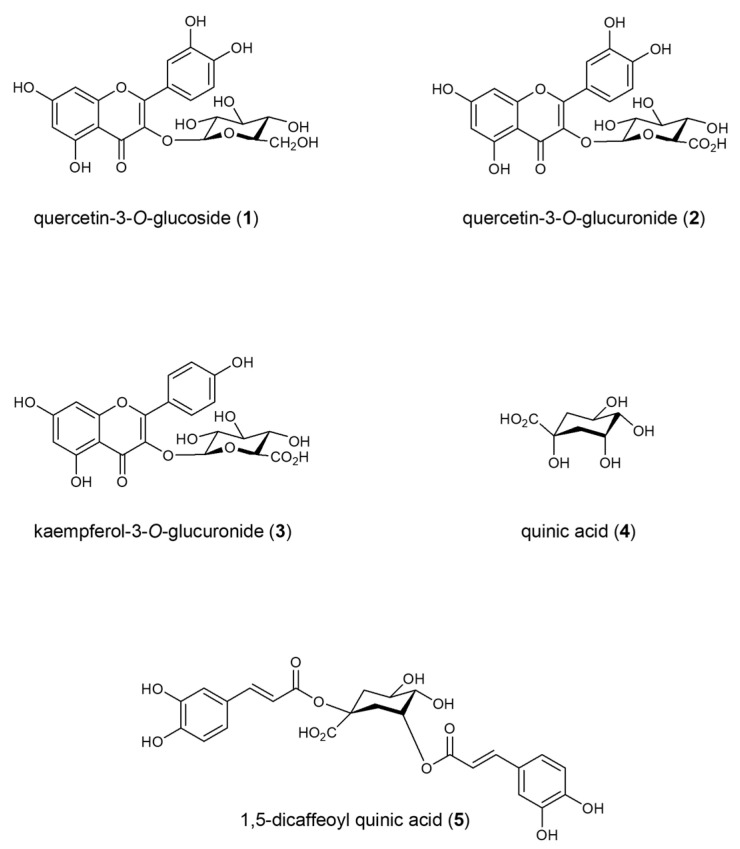
Structure of isolated compounds from *F. vulgare* waste.

The extract from fennel waste was separated into 15 fractions, and several compounds were isolated. Based on previously published work, the most abundant compounds in fennel waste were selected for the cell study [15]. The isolated compounds were identified by LC-MS and their structures were elucidated by NMR (nuclear magnetic resonance) spectroscopic data (Figure 1). Spectroscopic and spectrometric data, in addition, were compared with data obtained from market standards (Sigma-Aldrich) and literature data for final confirmation. 

Isolated compounds, with a purity level of 98% (LC-MS analysis), were identified as **1** (quercetin-3-*O*-glucoside) [16], **2** (quercetin-3-*O*-glucuronide) [17], **3** (kaempferol-3-*O*-glucuronide) [17], **4** (quinic acid) [18], and **5** (1,5-dicaffeoylquinic acid) [19].

### 2.2. UPLC–ESI-QTRAP-MS/MS Analyses of Eicosanoids and Fatty Acids in MRM (Multiple Reaction Monitoring) Mode

A method for the quantification of eicosanoids and fatty acids was developed using a QTrap 6500+ instrument (ABSciex, Foster City, CA, USA). The procedure for validation of the method was carried out according to the European Medicines Agency guidelines (EMA quality guidelines ICH Q2) [20], and is described in the supplement. The limit of detection (LOD) ranged from 0.006 to 0.02 ng/mL, and the limit of quantification (LOQ) ranged from 0.006 to 0.01 ng/mL. The correlation coefficients obtained were between 0.996 and 0.999.

To select the source parameters and MRM transitions, a literature review was conducted on studies that quantified eicosanoids and fatty acids using a QTrap mass spectrometer coupled with LC. Attention was focused on several key papers [21,22] and the chosen source parameters resulted in an intermediate between those proposed in the literature. The MS/MS parameters, such as collision energy, declustering potential, entrance potential, and collision cell potential, were taken from the literature [21,22]. Over 200 eicosanoids and fatty acids were searched for. The material and methods section shows the parameters chosen for the analysis of the eicosanoids and fatty acids detected in the samples. Only relevant data are shown, with the others reported in the Supplementary Materials (Table S1).

Before beginning the extraction and analysis of eicosanoids and fatty acids from the samples, preliminary tests were performed on the eicosanoid and fatty acid mixtures to ensure that the method could accurately detect them. Two issues emerged from these tests. The first was that the signal intensity was very low, especially for certain metabolites. This issue was addressed by increasing the injection volume from 2 μL to 7.5 μL. The second issue was that some eicosanoids, specifically leukotrienes, were not detected. Therefore, the method was optimized for leukotrienes. After searching METLINE (https://www.metline.eu/ accessed on 15 April 2022), additional transitions for leukotrienes, which were not originally included in the MRM list, were incorporated. However, this adjustment allowed for the detection of LTD4 only, while other leukotrienes remained undetected.

In the literature, leukotrienes have often been analyzed by mass spectrometry in positive ion mode [23]. For this reason, the polarity of the method was changed from negative to positive for the analysis of leukotrienes. Different collision energies (10, 15, 20, 25, and 30) were tested to further increase the intensity of the leukotrienes. The optimal collision energy (CE) was selected, which provided the most intense peak for leukotrienes.

**Figure 2:**
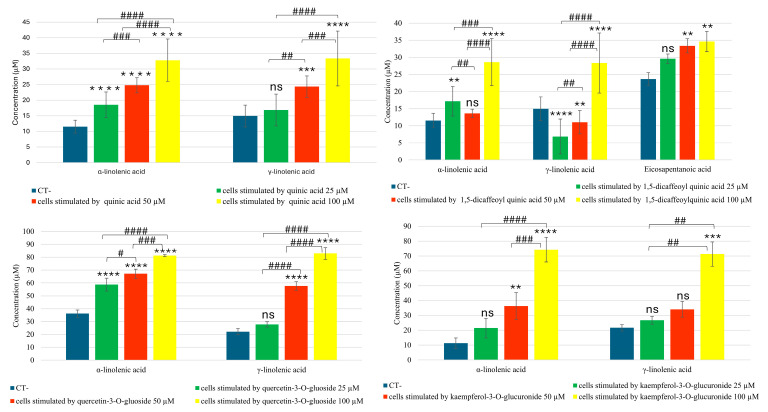
Targeted quantitative analysis of eicosanoids and fatty acids in BEAS-2B cells stimulated by quinic acid, 1,5-dicaffeoyl quinic acid, quercetin-3-*O*-glucoside and kaempferol-3-*O*-glucuronide by UHPLC-ESI-QTrap-MS/MS analysis in MRM (Multiple Reaction Monitoring) mode. A One-Way ANOVA was conducted for statistical analysis, followed by the Bonferroni multiple comparisons test. Values are expressed as mean ± SEM. ** *p* value < 0.005, *** *p* value < 0.001, **** *p* value < 0.0001 compared to the control. # *p* value < 0.05, ## *p* value < 0.005, ### *p* value < 0.001, #### *p* value < 0.0001 for the indicated concentration ranges. ns: statistically not significant.

Among the data obtained, in bronchial epithelial cells stimulated with quinic acid, there was a dose-dependent increase in alpha- and gamma-linolenic acids, two fatty acids with anti-inflammatory activity (Figure 2). Additionally, when either quercetin-3-*O*-glucuronide or kaempferol-3-*O*-glucuronide were added to the cells, a dose-dependent increase in these two fatty acids was observed. Specifically, treatment with these two flavonoids at the highest concentration of 100 µM resulted in an increase of more than 50% in both alpha- and gamma-linolenic acid.

Cells stimulated with 1,5-dicaffeoylquinic acid not only showed an increase in the two linolenic acids, but also an increase in eicosapentaenoic acid compared to the negative control (Figure 2).

The dose–response effect of the treatments was statistically significant in most cases, as shown in Figure 2.

In addition, treatment of the cells with 1,5-dicaffeoylquinic acid, quercetin-3-*O*-glucuronide, quercetin-3-*O*-glucoside, and kaempferol-3-*O*-glucuronide also increased another anti-inflammatory compound, docosahexaenoic acid (DHA) (Table 1). This molecule, while produced in minute quantities by the negative controls, reached concentrations of approximately one-hundred micromolar in cells stimulated with fennel metabolites at the highest concentration. DHA is a precursor of endogenous pro-resolving lipid mediators that regulate leukocyte trafficking and recruitment, thereby facilitating efferocytosis. Therefore, DHA may have a potential preventive role in the treatment of human cancer, which often arises due to an impaired resolution of inflammation and chronic inflammation [24] (Table 1). The statistical analysis of the dose–response effect of treatments on DHA increase is shown in Table S2.

Another result of this investigation was an increase in 20-COOH-LTB4, which was found to inhibit all the responses induced by leukotriene B4 (LTB4) in innate immunity, such as the recruitment of phagocytes, the release of antimicrobial effectors, and the enhancement of pathogen ingestion and killing [25]. Thus, 20-COOH-LTB4 exhibits anti-inflammatory activity by limiting the pro-inflammatory responses of LTB4.

Treatment with quercetin-3-*O*-glucuronide, quercetin-3-*O*-glucoside, kaempferol-3-*O*-glucuronide, and 1,5-dicaffeoylquinic acid induces an increase in 20-COOH-LTB4, with the strongest increase observed following stimulation of the cells with 1,5-dicaffeoylquinic acid. While a concentration of 64.55 µM of 20-COOH-LTB4 was observed in the negative control, the addition of 1,5-dicaffeoylquinic acid (100 µM) to BEAS-2B cells resulted in a concentration of 517.71 µM (Table 2). The statistical analysis of the dose–response effect of treatments on the increase of 20-COOH-LTB4 is shown in Table S2.

**Figure 3:**
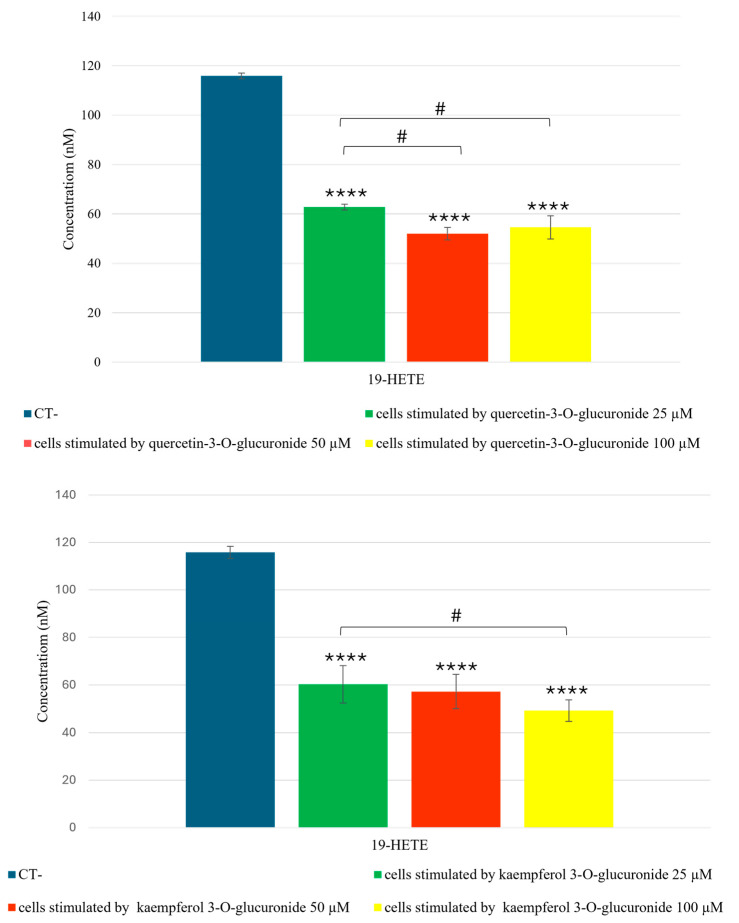
Targeted quantitative analysis of 19-HETE (19-hydroxyeicosatetraenoic acid) in BEAS-2B cells stimulated by quercetin 3-*O*-glucuronide and kaempferol 3-*O*-glucuronide, by UHPLC-ESI-QTrap-MS/MS analysis in MRM (Multiple Reaction Monitoring) mode. A One-Way ANOVA was conducted for statistical analysis, followed by the Bonferroni multiple comparisons test. Values are expressed as mean ± SEM. **** *p* value < 0.0001 compared to the control. # *p* value < 0.05, for the indicated concentration ranges.

In addition to inducing an increase in metabolites with anti-inflammatory activity, the glucuronic flavonoids identified in fennel waste reduce the levels of the pro-inflammatory eicosanoid 19-HETE (19-hydroxyeicosatetraenoic acid) by approximately 50% (Figure 3). This suggests that fennel waste may help reduce the inflammatory state in the human bronchial epithelium.

The dose–response effect for quercetin-3-*O*-glucuronide was statistically significant for the concentration ranges 100 µM vs. 25 µM and 100 µM vs. 50 µM. In contrast, for kaempferol-3-*O*-glucuronide, only the concentration interval 100 µM vs. 25 µM was statistically significant.

### 2.3. UPLC–ESI-QTRAP-MS Analyses of Sphingolipids in MRM (Multiple Reaction Monitoring) Modality

A method was optimized for the QTrap 6500+ mass spectrometer to analyze sphingolipids. The procedure for method validation is provided in the Supplementary Materials. The LOD (limit of detection) values ranged from 0.008 to 0.04 ng/mL, while the LOQ (limit of quantification) values ranged from 0.008 to 0.02 ng/mL. The correlation values obtained were between 0.996 and 0.999.

The method optimization was straightforward and performed on a standard mixture. Source and MS/MS parameters were adapted from a method already used in the Metabolomics Unit at the University of Lausanne [26,27]. Different collision energies were tested only for the sphingosine and sphinganine groups, which were initially not detected. For each metabolite, the best collision energy producing the highest intensity was selected. For other sphingolipid groups, such as ceramides, ceramide phosphates, and hexosylceramides, additional transitions were included, corresponding to the precursor ion's mass with the loss of a water molecule. This ensured the detection of all sphingolipids present in the mixture. Over 100 sphingolipids were analyzed. Table 3 shows the parameters used for analyzing the sphingolipids detected in the samples. Only relevant data are presented.

**Figure 4:**
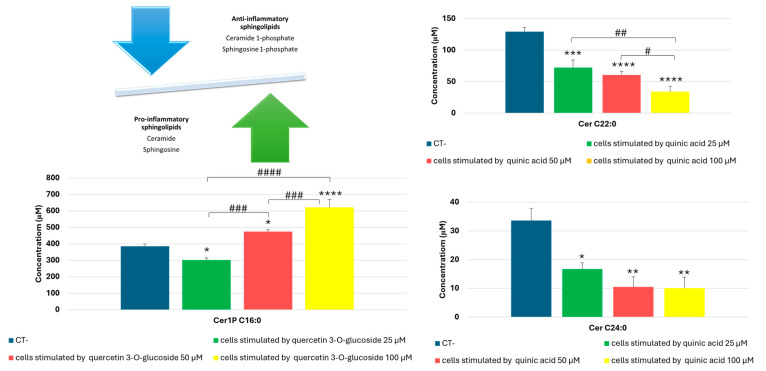
Targeted quantitative analysis of sphingolipids in BEAS-2B cells stimulated by quercetin 3-*O*-glucoside and quinic acid, by UHPLC-ESI-QTrap-MS/MS analysis in MRM (Multiple Reaction Monitoring) mode. A One-Way ANOVA was conducted for statistical analysis, followed by the Bonferroni multiple comparisons test. Values are expressed as mean ± SEM. * *p* value < 0.05, ** *p* value < 0.005, *** *p* value < 0.001, **** *p* value < 0.0001 compared to the control. # *p* value < 0.05, ## *p* value < 0.005, ### *p* value < 0.001, #### *p* value < 0.0001 for the indicated concentration ranges. Cer C22:0, Ceramide C22:0; Cer1P C16:0, Ceramide 1-phosphate C16:0; Cer C24:0, Ceramide C24:0.

Regarding the data obtained from the quantitative analysis of sphingolipids, human bronchial epithelial cells stimulated with quinic acid showed a dose-dependent decrease in two pro-inflammatory ceramides: ceramide C22 and ceramide C24. Furthermore, treatment of bronchial epithelial cells with quercetin-3-*O*-glucoside led to an increase in ceramide 1-phosphate C16, which was also directly proportional to the concentration of quercetin-3-*O*-glucoside (Figure 4). The dose–response effect of CER C24:0 reduction following quinic acid treatment was not statistically significant.

Thus, it has been shown that the main metabolites of fennel waste can help to restore the balance between the expression of some anti-inflammatory and pro-inflammatory sphingolipids.

## 3. Discussion

*F. vulgare* cultivation generates a large amount of waste. Previous studies have shown that these wastes are rich in bioactive compounds such as phenolic acids and glycosylated flavonoids. In addition to incorporating parts of fennel that are not typically used in cooking, such as leaves and stems, it is of interest to utilize them in the production of functional foods or nutraceutical products.

For example, anti-inflammatory nutraceuticals are widely used in the treatment of chronic inflammatory conditions such as asthma and myalgia, where the long-term use of anti-inflammatory drugs can lead to significant side effects.

Following preliminary studies to evaluate the potential biological activities of the metabolites most abundant in fennel [12], we proceeded with an in vitro study on human bronchial epithelial cells. After inducing inflammation in the cells and stimulating them with IL-1β, they were treated with various metabolites isolated from fennel waste at different concentrations. To assess whether these metabolites could alleviate the inflammatory state in the cells, two classes of metabolites involved in the regulation of inflammatory processes—eicosanoids and sphingolipids—were quantified.

Metabolomic experiments using mass spectrometry in this investigation showed that the natural compounds in fennel waste, such as quinic acid, quercetin 3-*O*-glucoside, kaempferol 3-*O*-glucuronide, and 1,5-dicaffeoyl quinic acid, induce the production of fatty acids with anti-inflammatory activity, such as α- and γ-linolenic acids. Therefore, the inflammatory states associated with chronic inflammatory diseases can be alleviated by increasing molecules with anti-inflammatory properties. Indeed, it has been demonstrated in the literature that α-linolenic acid can reduce the inflammatory state of colitis by down-regulating the mRNA levels of several pro-inflammatory genes, such as IL-6, cyclooxygenase 2, and tumor necrosis factor α [28]. Furthermore, the metabolites isolated from fennel waste in this study (kaempferol-3-*O*-glucuronide and quercetin 3-*O*-glucuronide) can improve the inflammatory state in the human bronchial epithelium by reducing the levels of 19-HETE eicosanoid, which is known to induce and exacerbate inflammation. The specialized fennel metabolites, except for kaempferol-3-*O*-glucuronide, also cause an increase in 20-COOH-LTB4, which inhibits the pro-inflammatory leukotriene LTB4 response, such as chemotactic activity on eosinophils.

Plant metabolites can reduce the production of linolenic acid in the human body through various mechanisms. One such mechanism is the reduction in oxidative stress via their antioxidant activity [29]. Oxidative stress plays a key role in the regulation of lipid metabolism and can influence linolenic acid synthesis [30]. Natural substances neutralize reactive oxygen species (ROS), which may otherwise reduce the activity of lipid enzymes involved in the biosynthesis of pro-inflammatory molecules, such as delta-6-desaturase (D6D) and delta-5-desaturase (D5D). Given previous studies demonstrating the antioxidant activity of fennel waste [15], it is plausible that natural compounds isolated from this waste could help reduce inflammation in the human bronchial epithelium by mitigating oxidative stress.

It has been shown that in various inflammatory diseases, such as COPD, there is an imbalance between pro-inflammatory and anti-inflammatory sphingolipids [22]. In particular, there is an increase in ceramides and a decrease in ceramide phosphates, which instead help reduce inflammation. Quantitative analysis of sphingolipids in this research showed that fennel metabolites balance the expression of pro- and anti-inflammatory sphingolipids. Quinic acid, for example, down-regulates the levels of ceramides C22 and C24, which are pro-inflammatory sphingolipids. On the other hand, quercetin 3-*O*-glucoside increases the levels of ceramide 1-phosphate C16, which has anti-inflammatory activity.

## 4. Materials and Methods

### 4.1. Plant Material and Isolation Procedure

Fennel waste of Pegaso variety, harvested in April 2021, was provided by the Paolillo company (Salerno-Italy). 150 g of fennel waste was extracted with a solution of ethanol/water (80/20) (VWR) for 3 days. After filtration and evaporation of the solvent to dryness in vacuo, 38 g of hydroalcoholic extract was obtained.

The hydroalcoholic extract was dried under vacuum and 3 g were fractionated on a Sephadex LH-20 (Pharmacia) column (100 × 5 cm), using MeOH (VWR) as mobile phase, affording 74 fractions (8 mL). After monitoring by TLC, equal fractions were combined to form 15 fractions.

Semi-preparative HPLC (Agilent, Milan, Italy) chromatographed the fractions. The mobile phases used were water +0.1% acetic acid (A) (VWR) and Acetonitrile +0.1% acetic acid (B) (VWR). The increasing linear-gradient (*v*/*v*) at a flow rate of 2.0 mL/min of solvent B was used: 0–10 min, from 5 to 15%; 10–30 min, from 15 to 35%; 30–40, from 35 to 80%, and then back to 5% for 10 min. A Phenomenex Luna C18 5 μm (250 × 10.00 mm) column (Phenomenex Aschaffenburg, Germany) was used. The structural characterization was carried out by nuclear magnetic resonance experiments 1D-NMR and 2D-NMR.

### 4.2. Cell Experiments

The BEAS-2B line (human bronchial epithelial cells) purchased from the American Type Culture Collection (ATCC, Massan, VA, USA) was used. The line consists of non-cancerous bronchial epithelial cells obtained from healthy individuals, immortalized through transformation with the SV40 adenovirus. Cells were maintained in growth medium composed of Ham’s F12/DMEM (EuroClone), L-glutamine (2 mM) (Lonza), penicillin (100 U/mL), and streptomycin (100 mg/mL) (Lonza), supplemented with 5% Fetal Bovine Serum (FBS, EuroClone), previously subjected to thermal inactivation and kept at a temperature of 37 °C and in the presence of 5% CO_2_.

Cells were seeded at 50,000 cells/well in 48-well plates, and after 24 h the medium were replaced with fresh medium (FBS 1%) for another 24 h. Cells were then treated with different polyphenol metabolites (quercetin 3-*O*-glucoside, quercetin 3-*O*-glucuronide, kaempferol 3-*O*-glucuronide, 1,5-dicaffeoylquinic acid, and quinic acid) at different concentrations (25, 50, 100 μM) in triplicate and co-stimulated with 1 ng/mL of IL-1β for 18 h. The metabolites were dissolved in DMSO and then diluted into different concentrations with a culture medium. The final DMSO concentration in all assays did not exceed 0.1% (*v*/*v*). Cells without treatment served as a negative control. After 18 h, the medium of the cells was collected and stored at −80 °C. The cells were counted after the various cell treatments, and their viability was verified with the trypan blue exclusion test (EuroClone). Cell viability after treatment with fennel waste metabolites was ≥97% at harvest in all conditions (Table S3).

### 4.3. Eicosanoids and Fatty Acids Extraction

The eicosanoids were isolated and quantified in the University of Lausanne using a metabolomic platform. The eicosanoids were extracted from the cell medium via solid phase extraction (SPE). Before SPE, standard solutions were prepared at 13 different concentrations, and a buffer solution was prepared by mixing 58 mL of 0.2 M Na_2_HPO_4_ with 42 mL of 0.1 M citric acid. A 96 multi-well was prepared with wells for calibrators and wells for samples.

A total of 70 μL of calibrator, 10 μL of ISTD, and 920 μL of buffer were added in the calibrator wells. In the sample wells, 500 μL of cell culture media, 10 μL of ISTD, and 490 μL of buffer were added. The multi-well was vortexed for 5 min at 120 rpm.

For SPE, the multi-well was conditioned with methanol at 7 psi for 1 min + 3 times for 2 s at 40 psi. After, it was equilibrated with water at 7 psi for 1 min + 3 times for 2 s at 40 psi. The samples were loaded onto the SPE plate at 4 psi for 3 min and at 30 psi for 30 s. The SPE plate was washed with a solution of 4:1 H_2_O/MeOH at 4 psi for 2 min + 3 times for 2 s at 40 psi. After, it was dried at 5 psi for 2 min and at 30 psi for 14 min. Finally, the elution was performed with 750 μL of MeOH for 1 min and then at 2 psi for 4 min and at 30 psi for 2 min, and this was done two times. The solvent was evaporated using a Turbovap (Biotage, Uppsala, Sweden). The samples were so reconstituted in 75 μL of 85.7% of MeOH with H_2_O.

### 4.4. UPLC–ESI-QTRAP-MS Analyses of Eicosanoids and Fatty Acids in MRM (Multiple Reaction Monitoring) Modality

Quantitative analysis of the eicosanoids extracted from cell media of BEAS-2B cell line was carried out with a Q-TRAP 6500+ LC-MS and an LC-MS/MS (ABSciex, Foster City, CA, USA) equipped with an Acquity UPLC BEH C18 1.7 μm column and a VanGuard Acquity UPLC BEH C18 1.7 μm pre-column from Waters (Milford, MA, USA) [31]. The mobile phases used were water +0.1% acetic acid (A) and Acetonitrile/Isopropanol (9/1) (B). The metabolites were chromatographically separated using the following increasing linear-gradient (*v*/*v*) at a flow rate of 0.500 mL/min of solvent B: 0.1–2.50 min, from 20 to 35%; 2.50–4.50 min, from 35 to 40%; 4.50–6.00 min, from 40 to 42%; 6.00–8.00, from 42 to 50%; 8.00–14.00 min, from 50 to 65 %; 14.00–15.50 min, from 65 to 72.5 %; 15.50–16.1 min, from 72.5 to 100%; and then back to 20% for 3.40 min. The ion mode was negative and 7.5 μL of each sample was used for injection. The 6500+ QTRAP was set up for IonSpray operation, and the compounds were detected using multiple reaction monitoring (MRM) (Table 3). Mass spectrometry source parameters were set up as follows: curtain gas (CUR) = 30; collision gas (CAD) = medium; ion spray voltage (IS) = −4200; temperature (TEM) = 520; ion source gas 1 (GS1) = 85; ion source gas 2 (GS2) = 50. The dwell time for each analyte was 20 ms. Analyst software 1.6.2 was used for data acquisition. Multiquant software 3.0.3 was used for processing (ABSciex, Foster City, CA, USA). Data processed with Multiquant software were analyzed using a one-way ANOVA followed by the Bonferroni multiple comparisons test with GraphPad Prism 10 software (GraphPad Software Inc., Boston, MA, USA).

### 4.5. Sphingolipids Extraction

The sphingolipids were isolated and quantified in the University of Lausanne at Metabolomic Platform. Sphingolipids calibration solutions were prepared at eleven concentrations. An internal standard mixture in MeOH 100% was prepared by mixing 400 μL of solution of internal standard and 5600 μL of MeOH. In the multi-wells, 100 μL of ice-cold methanol internal standard mix and 25 μL of cell media or calibrator solutions were added. The extraction was performed by Robot Bravo (Agilent, Milan, Italy). 

### 4.6. UPLC–ESI-QTRAP-MS Analyses of Sphingolipids in MRM (Multiple Reaction Monitoring) Modality

The analysis of the sphingolipids extracted from cell media of BEAS-2B cell line was carried out with a Q-TRAP 6500+ from Sciex LC-MS and an LC-MS/MS equipped with a column Zorbax Eclipse Plus C8 (100 mm × 2.1 mm ID, 1.8 μm) [32]. The mobile phases used were 5 mM Ammonium Formate, 0.2 % (*v*/*v*) formic acid in water (A), and 5 mM Ammonium Formate, 0.2 % (*v*/*v*) formic acid in methanol (B). The metabolites were chromatographically separated using the following increasing linear-gradient (*v*/*v*) at a flow rate of 0.400 mL/min of solvent B: 0.1–8.0 min, from 80 to 100%; 8.0–14.5 min, from 100 to 80% until the stop of the course at 20 min. The ion mode was positive and 2 μL of each sample was used for injection. The 6500+ QTRAP was set up for IonSpray operation, and the compounds were detected using multiple reaction monitoring (MRM) (Table 4). Mass spectrometry source parameters were set up as follows: curtain gas (CUR) = 35; collision gas (CAD) = medium; ion spray voltage (IS) = 5500; temperature (TEM) = 550; ion source gas 1 (GS1) = 50; ion source gas 2 (GS2) = 60. The dwell time for each analyte was 20 ms. Analyst software 1.6.2 was used for data acquisition. Multiquant software 1.6.2 was used for processing (ABSciex, Foster City, CA, USA). Data processed with Multiquant software were analyzed using a one-way ANOVA followed by the Bonferroni multiple comparisons test with GraphPad Prism 10 software (GraphPad Software Inc.)

## 5. Conclusions

There is growing interest in the utilization of plant waste. In recent years, scientific research has explored new applications for bioactive compounds derived from plant by-products. This study is set within this context. Fennel production generates large quantities of by-products, and, notably, the least-used parts of fennel in cooking are the richest sources of compounds with significant biological properties. Using an innovative metabolomic approach based on mass spectrometry, we have demonstrated that metabolites extracted from fennel by-products possess strong anti-inflammatory potential in the human bronchial epithelium. Specifically, they can reduce the expression of pro-inflammatory molecules while simultaneously inducing the production of anti-inflammatory molecules. As a result, this action leads to a reduction in the inflammatory state of the human bronchial epithelium. From the results of the studies carried out on human bronchial epithelial cells, it can be concluded that fennel waste, thanks to its content of bioactive compounds, can be used in the production of nutraceutical products which will be particularly useful in the prevention or treatment of respiratory tract pathologies. These products, derived from foods with bioactive properties, can modulate inflammation and oxidative stress which are two key factors in the development and progression of diseases such as asthma, chronic obstructive pulmonary disease (COPD), and idiopathic pulmonary fibrosis (IPF).

## Figures and Tables

**Table 1:** Targeted quantitative analysis of docosahexaenoic acid (DHA) in BEAS-2B cells stimulated by kaempferol-3-*O*-glucuronide, quercetin-3-*O*-glucuronide, quercetin-3-*O*-glucoside and 1,5-dicaffeoyl quinic acid by UHPLC-ESI-QTrap-MS/MS analysis in MRM (Multiple Reaction Monitoring) mode.

**Cells Stimulated by Kaempferol 3-*O*-glucuronide**			
CT-	25 µM	50 µM	100 µM
8.6 ± 0.5	58 ± 7 (**)	75.3 ± 6.0 (***)	106 ± 9 (***)
Cells stimulated by quercetin 3-*O*-glucuronide			
CT-	25 µM	50 µM	100 µM
8.6 ± 0.5	49.4 ± 2.6 (ns)	55.6 ± 1.7 (*)	71.8 ± 4.9 (**)
Cells stimulated by quercetin 3-*O*-glucoside			
CT-	25 µM	50 µM	100 µM
12.8 ± 3.4	69.5 ± 2.8 (****)	83.2 ± 5.8 (****)	107.4 ± 2.5 (****)
Cells stimulated by 1,5-dicaffeoylquinic acid			
CT-	25 µM	50 µM	100 µM
7.2 ± 0.7	51.91 ± 0.56 (**)	114.0 ± 1.5 (****)	140.9 ± 1.3 (****)

The data represent the average of three experiments, expressed as concentration µM of DHA in cell media ± standard deviation. A One-Way ANOVA was conducted for statistical analysis, followed by the Bonferroni multiple comparisons test. * *p* value < 0.05, ** *p* value < 0.005, *** *p* value < 0.001, **** *p* value < 0.0001 compared to the control, ns: statistically not significant.

**Table 2:** Targeted quantitative analysis of 20-COOH-LTB4 in BEAS-2B cells stimulated by quercetin-3-*O*-glucuronide, quercetin-3-*O*-glucoside, quinic acid and 1,5-dicaffeoyl quinic acid by UHPLC-ESI-QTrap-MS/MS analysis in MRM (Multiple Reaction Monitoring) mode.

**Cells Stimulated by Quercetin 3-*O*-glucuronide**			
CT-	25 µM	50 µM	100 µM
171.9 ± 8.6	208.7 ± 8.1 (**)	198 ± 18 (*)	301.2 ± 4.9 (****)
Cells stimulated by quercetin 3-*O*-glucoside			
CT-	25 µM	50 µM	100 µM
56.1 ± 6.9	82.4 ± 6.4 (*)	83 ± 13 (*)	100 ± 15 (***)
Cells stimulated by quinic acid			
CT-	25 µM	50 µM	100 µM
64.6 ± 7.9	74.5 ± 6.2 (ns)	101 ± 18 (**)	91.86 ± 1.49 (*)
Cells stimulated by 1,5-dicaffeoyl quinic acid			
CT-	25 µM	50 µM	100 µM
64.6 ± 7.9	344.0 ± 19.8 (****)	415.7 ± 5.9 (****)	518 ± 30 (****)

The data represent the average of three experiments, expressed as concentration µM of 20-COOH-LTB4 in cell media ± standard deviation. A One-Way ANOVA was conducted for statistical analysis, followed by the Bonferroni multiple comparisons test. * *p* value < 0.05, ** *p* value < 0.005, *** *p* value < 0.001, **** *p* value < 0.0001 compared to the control, ns: statistically not significant.

**Table 3:** Mass spectral parameters and precursor/product MRM transitions of standard eicosanoids and fatty acids measured using a UHPLC system coupled to an ABSciex Q-Trap 6500+ instrument in MRM mode (UPLC-ESI-QTRAP-MS/MS).

Eicosanoids and Fatty Acids	DP	EP	CE	CXP	Precursor	Product
α-Linolenic acid	−30	−10	−30	−10	277.2	127.1
γ-Linolenic acid	−30	−10	−40	−10	277.2	191.1
Linolenic acid	−30	−10	−20	−10	279.2	261.2
Eicosapentanoic acid	−40	−10	−16	−10	301.2	257.2
Arachidonic acid	−55	−10	−20	−10	303.2	259.2
Docohexaenoic acid	−40	−10	−19	−10	327.3	283.2
5-HETE	−40	−10	−20	−10	319.1	114.9
9-HETE	−40	−10	−20	−10	319.1	179.1
12-HETE	−50	−10	−19	−10	319.0	178.8
15-HETE	−30	−10	−17	−10	319.1	174.8
19-HETE	−40	−10	−23	−20	319.2	275.1
20-HETE	−50	−10	−24	−20	319.2	274.9
9,10,13-TriHOME	−50	−10	−29	−10	329.0	139.0
9,12,13-TriHOME	−50	−10	−29	−10	329.1	211.0
11-HDoHE	−20	−10	−19	−10	343.3	149.0
14-HDoHE	−30	−10	−19	−10	343.2	281.2
17-HDoHE	−30	−10	−19	−10	343.2	281.2
12,13-DiHOME	−50	−10	−29	−10	313.1	183.1
11(12)-EpETrE	−40	−10	−20	−20	319.2	166.9
11,12-DiHETrE	−40	−10	−25	−10	337.2	167.0
14,15-DiHETrE	−30	−10	−24	−10	337.2	207.0
LTB4	−45	−10	−23	−10	335.2	195.0
20-COOH-LTB4	−40	−10	−26	−10	365.2	195.1
TXB2	−50	−10	−35	−10	369.2	168.9
TXB3	−40	−10	−27	−10	367.2	195.2
11-keto TXB3	−40	−10	−26	−10	365.2	169.2
tetranor-PGDM	−30	−10	−23	−10	327.1	155.0
PGB2	−40	−10	−30	−10	333.2	175.0
6-keto-PGF1a	−60	−10	−34	−10	369.2	162.9

DP Declustering potential, EP Entrance Potential, CE Collision energy, CXP Collision Cell Exit Potential.

**Table 4:** Mass spectral parameters and precursor/product MRM transitions of standard sphingolipids measured using a UHPLC system coupled to an ABSciex Q-Trap 6500+ instrument in MRM mode (UPLC-ESI-QTRAP-MS/MS).

Sphingolipids	DP	EP	CE	CXP	Precursor	Product
GlcCer C16:0 (d18:1/16:0)	60	10	43	15	682.6	520.5
GlcCer C24:1 (d18:1/24:1(15Z)) (1)	60	10	43	15	792.7	612.6
GlcCer C24:1 (d18:1/24:1(15Z)) (2)	60	10	43	15	792.7	264.3
LacCer C24:1 (d18:1/24:1) (1)	60	10	43	15	954.7	264.3
LacCer C24:1 (d18:1/24:1) (2)	60	10	43	15	954.7	630.6
N-lauroyl-1-deoxysphingosine (1)	60	10	43	15	466.5	448.4
N-lauroyl-1-deoxysphingosine (2)	60	10	43	15	466.5	266.2
N-nervonoyl-1-deoxysphinganine	60	10	43	15	634.7	616.5
SM C12:0 (d18:1/12:0)	60	10	43	15	647.5	184.1
SM C16:0 (d18:1/16:0)	60	10	43	15	703.6	184.1
SM C18:0 (d18:1/18:0)	60	10	43	15	731.6	184.1
SM C18:1 (d18:1/18:1(9Z))	60	10	43	15	729.6	184.1
SM C24:0 (d18:1/24:0)	60	10	43	15	815.7	184.1
SM C24:1 (d18:1/24:1(15Z))	60	10	43	15	813.7	184.1
SM C6:0 (d18:1/6:0)	60	10	43	15	563.4	184.1
Spa 1P (d18:0)	60	10	20	15	382.3	95.1
Sph (d20:1)	60	10	43	15	310.3	81.1
1-deoxysphingosine	60	10	43	15	284.3	266.2
C18 Dihydroceramide	60	10	43	15	568.6	524.5
C20 Dihydroceramide	60	10	43	15	596.6	298.1
C22 Sphingomyelin (1)	60	10	43	15	787.7	184.0
C22 Sphingomyelin (2)	60	10	43	15	787.7	86.1
Cer C18:1 (d18:1/18:1(9Z)	60	10	43	15	546.5	237.1
Cer C2:0 (d18:1/2:0)	60	10	43	15	324.3	306.3
1-desoxymethylsphingosine	60	10	43	15	270.3	252.2
Cer C24:0 (d18:1/24:0)	60	10	43	15	632.6	264.3
Cer C20:0 (d18:1/20:0)	60	10	43	15	576.6	264.3
Cer1P C16:0 (d18:1/16:0)	60	10	43	15	600.5	520.5
Cer1P C18:1 (d18:1/18:1(9Z))	60	10	43	15	644.5	264.3
DhCer C24:0 (d18:0/24:0) (1)	60	10	43	15	652.7	634.6
DhCer C24:0 (d18:0/24:0) (2)	60	10	43	15	652.7	302.3
DhCer C24:0 (d18:0/24:0) (3)	60	10	43	15	652.7	284.3
DhCer C24:1 (d18:0/24:1(15Z)) (1)	60	10	43	15	650.6	632.6
DhCer C24:1 (d18:0/24:1(15Z)) (2)	60	10	43	15	650.6	302.3

DP Declustering potential, EP Entrance Potential, CE Collision energy, CXP Collision Cell Exit Potential.

## Data Availability

The data presented in this study is contained within the article.

## Supplementary Materials

The following supporting information can be downloaded at: www.mdpi.com/article/10.3390/molecules30071407/s1, Method validation. Table S1. Targeted quantitative analysis of eicosanoids in cells stimulated by kaempferol-3-*O*-glucuronide, quercetin-3-*O*-glucuronide, quercetin-3-*O*-glucoside, quinic acid and 1,5-dicaffeoyl quinic acid by UHPLC-ESI-QTrap-MS/MS analysis in MRM mode. (Data not relevant). Table S2. Results of One-Way ANOVA test conducted for statistical analysis, followed by the Bonferroni multiple comparisons test. # *p* value < 0.05, ## *p* value < 0.005, ### *p* value < 0.001, #### p value < 0.0001 for the indicated concentration ranges. ns: statistically not significant. DHA (docosahexaenoic acid). Table S3. Viability of BEAS-2B cells stimulated with fennel waste metabolites, evaluated with Trypan Blue exclusion test.
