# Supplementary material for: A Targeted Mass Spectrometric Approach to Evaluate the Anti-Inflammatory Activity of the Major Metabolites of Foeniculum vulgare Mill. Waste in Human Bronchial Epithelium
Source: Molecules. 2025 Mar 21;30(7):1407. doi: 10.3390/molecules30071407 (PMC11990374; doi:10.3390/molecules30071407)
Supplement: Supplementary file 1 [file molecules-30-01407-s001.zip › molecules-3464885-supplementary.pdf]

Article

A Targeted Mass Spectrometric Approach to Evaluate the Anti-Inflammatory Activity of the Major Metabolites of *Foeniculum vulgare* Mill. Waste in Human Bronchial Epithelium

Maria Assunta Crescenzi [1,2], Hector Gallart-Ayala [3], Cristiana Stellato [4], Ada Popolo [1], Julijana Ivanisevic [3], Sonia Piacente [1] and Paola Montoro [1,*]

[1] Department of Pharmacy, University of the Study of Salerno, Via Giovanni Paolo II 132, I-84084 Fisciano, Italy
[2] Ph.D. Program in Drug Discovery & Development, Department of Pharmacy, University of the Study of Salerno, Via Giovanni Paolo II 132, I-84084 Fisciano, Italy
[3] Metabolomics Unit, Faculty of Biology and Medicine, University of Lausanne, 1015 Lausanne, Switzerland
[4] Department of Medicine, Surgery and Dentistry "Scuola Medica Salernitana", University of Salerno, 84084 Salerno, Italy
* Correspondence: pmontoro@unisa.it

Method validation

In order to validate the analytical methods, the UPLC-ESI-QTRAP-MS/MS method was validated according to the European Medicines Agency guidelines (EMA quality guidelines ICH Q2). The limit of quantification (LOQ; equivalent to sensitivity) was assessed by injecting a series of progressively diluted standard solutions until the signal-to-noise ratio was reduced to 10. The limit of detection (LOD) was estimated by injecting a series of increasingly diluted standard solutions until the signal-to-noise ratio was reduced to 3.

Table S1. Targeted quantitative analysis of eicosanoids in cells stimulated by kaempferol-3-*O*-glucuronide, quercetin-3-*O*-glucuronide, quercetin-3-*O*-glucoside, quinic acids and 1,5-dicaffeoyl quinic acid by UHPLC-ESI-QTrap-MS/MS analysis in MRM mode. (Data not relevant).

**Eicosanoids**	**Cells stimulated by kaempferol 3-*O*-glucuronide**			
	CT-	25 µM	50 µM	100 µM
eicosapentanoic acid	6.3±1.0	5.7±1.2	9.4±0.7	14.0±2.6
20-COOH LTB4	171.9±8.6	121±5	127±3	130±4
	**Cells stimulated by quercetin 3-*O*-glucuronide**			
	CT-	25 µM	50 µM	100 µM
α-linolenic acid	11.2±3.7	13.2±2.5	9.7±3.2	12.3±1.2
γ-linolenic acid	21.6±2.2	22.9±1.3	20±12	16.8±3.3
eicosapentanoic acid	6.3±1.0	3.5±0.4	4.0±1.2	5.2±1.7
	**Cells stimulated by quercetin 3-*O*-glucoside**			
	CT-	25 µM	50 µM	100 µM
eicosapentanoic acid	35.1±0.9	36.0±0.9	34.1±1.4	34.0±3.0

19-HETE	78.7±10.6	73.6±2.5	73.6±9.8	79.2±5.2
	**Cells stimulated by quinic acid**			
	CT-	25 µM	50 µM	100 µM
docohexaenoic acid	nd	nd	nd	nd
eicosapentanoic acid	23.7±1.8	27.4±1.8	27.9±1.9	27.8±1.3
19-HETE	63.5±1.6	70.5±3.5	65.5±6.2	66.4±2.7
	**Cells stimulated by 1,5-dicaffeoyl quinic acid**			
	CT-	25 µM	50 µM	100 µM
19-HETE	63.5±1.6	61.1±1.2	60.3±2.2	58.1±5.2

Table S2. Results of One-Way ANOVA test conducted for statistical analysis, followed by the Bonferroni multiple comparisons test. # p value < 0.05, ## p value < 0.005, ### p value < 0.001, #### p value < 0.0001 for the indicated concentration ranges. ns: statistically not significant. DHA (docosahexaenoic acid).

	Cells stimulated by kaempferol 3-*O*-glucuronide		
	25 µM vs 50 µM	25 µM vs 100 µM	50 µM vs 100 µM
DHA	ns	##	#
	Cells stimulated by quercetin 3-*O*-glucuronide		
DHA	ns	ns	ns
20-COOH-LTB4	ns	####	####
	Cells stimulated by quercetin 3-*O*-glucoside		
DHA	###	####	####
20-COOH-LTB4	ns	ns	ns
	Cells stimulated by quinic acid		
20-COOH-LTB4	#	ns	ns
	Cells stimulated by 1,5-dicaffeoyl quinic acid		
DHA	##	####	ns
20-COOH-LTB4	##	####	###

Table S3. Viability of BEAS-2B cells stimulated with fennel waste metabolites, evaluated with Trypan Blue exclusion test.

	Viability (%)
Control	98.4
Quinic acid 25 µM	97.3
Quinic acid 50 µM	96.6
Quinic acid 100 µM	99.0
1,5-Dicaffeoyl quinic acid 25 µM	99.3
1,5-Dicaffeoyl quinic acid 50 µM	94.0
1,5-Dicaffeoyl quinic acid 100 µM	99.1
Control	98.3
Quercetin 3-O-glucuronide 25 µM	98.2
Quercetin 3-O-glucuronide 50 µM	97.3
Quercetin 3-O-glucuronide 100 µM	99.5
Kaempferol 3-O-glucuronide 25 µM	99.3
Kaempferol 3-O-glucuronide 50 µM	98.8

Kaempferol 3-O-glucuronide 100 µM	97.5
Control	98.6
Quercetin 3-O-glucoside 25 µM	97.7
Quercetin 3-O-glucoside 50 µM	99.2
Quercetin 3-O-glucoside 100 µM	97.9
